# Bridging art and AI in the global south: the development of the robot Zequinha considering the grand challenges of human-centered artificial intelligence

**DOI:** 10.3389/frobt.2026.1765950

**Published:** 2026-02-25

**Authors:** Ana Claudia da Cunha, Elisa Granha Lira, Saulo José Dansa de Alencar, Roberto Bartholo, Heitor Mansur Caulliraux

**Affiliations:** Laboratory of Technologies, Dialogues and Sites (LTDS), Production Engineering Program, Alberto Luiz Coimbra Institute for Graduate Studies and Research in Engineering (COPPE), Universidade Federal do Rio de Janeiro, Rio de Janeiro, Brazil

**Keywords:** advanced technologies, artificial intelligence (AI), creative media, cultural heritage, extended reality (XR), HCAI challenges, human-AI interaction, human-centered artificial intelligence

## Abstract

This paper presents the design of a Brazilian robot, named Zequinha, for cultural and educational purposes in light of Human-Centered Artificial Intelligence challenges. Zequinha’s development is a blend of art, robotics, and AI, evolving from MIDI-programmed animatronics to an autonomous entity integrating multiple local AIs. This shift to local processing inherently enhances privacy and governance, minimizing reliance on external APIs and enabling offline operability. The project’s human-centered design approach is evident in its iterative methodology and its collaborative development with children. Zequinha promotes human wellbeing by enriching cultural mediation, engaging diverse audiences, and demonstrating potential in health and education. Moreover, the focus on local AI fosters responsible design and meaningful human-AI interaction, aiming to create a charismatic, safe, and useful robotic mediator.

## 1 Introduction

Museums worldwide have increasingly adopted virtual assistants based on natural language Artificial Intelligence (AI) to enhance visitors’ experience, often relying on Small Language Models (SLMs) trained using their own data (Capece et al., 2024). The UNESCO report “Small Language Models (SLMs): A Cheaper, Greener Route into AI” (Wang and Wang, 2024) highlights how well-trained small models can deliver customized solutions with significantly lower carbon footprints and costs, avoiding the inefficiency of using a sledgehammer to crack a nut—that is, deploying massive LLMs for tasks that SLMs can handle effectively. In other words, at the intersection of art, culture, and AI, SLMs are carving out a meaningful role, reinventing creativity and heritage experiences in accessible and innovative ways.

For example, SLMs can be used as cultural chatbots with a focused scope to offer interactive and educational experiences whose content is specifically tailored to the institution’s mission, without requiring the infrastructure or costs associated with Large Language Models (LLMs), such as ChatGPT (Caballar, 2024). These systems have been implemented as virtual assistants and chatbots that can answer questions, guide visitors, and contextualize collections, often through mobile devices or on-screen interfaces. Examples include AI-driven cultural mediators developed in collaboration with major technology providers. In Brazil, two pioneering cases illustrate the use of AI for cultural mediation, both developed in collaboration with IBM Brazil. The Pinacoteca of São Paulo implemented the “The Voice of Art” project in 2017, which enabled visitors to submit questions to a chatbot about artworks on display via their mobile phones and receive answers in natural language (Vicelli and Kunsch, 2024). Powered by the museum’s archives, as well as news sources and general internet content, the assistant could provide historical details about the paintings and even simulate conversations with the characters depicted in them (Gaia et al., 2019). For instance, when approaching a painting, visitors could ask, “Who is the person in this painting?” and the chatbot would respond with contextual information, often “speaking” as if it were the artwork itself or a historical artist. Behind the scenes, such a chatbot relies on focused natural language processing models—functionally equivalent to SLMs—combined with dialogue trees and curated knowledge bases to ensure reliability. Opting for this architecture, rather than a generic cloud-based LLM, offers clear advantages: preserving visitor data privacy, ensuring content control (responses are confined to validated collection data, minimizing the risk of hallucinations), and enabling stable operation even under offline conditions or unstable internet connectivity within the historical building.

Similarly, though more elaborately, IRIS+ (Morena, 2018a), the artificial intelligence system of the “Museu do Amanhã” (Rio de Janeiro), launched in 2018, was built upon IBM Watson AI services, which, while not equivalent to current large transformer-based LLMs, incorporate optimized language and dialogue models for specific domains. Integrated into the museum’s leading exhibition, IRIS + invites visitors to engage in dialogue: after the exhibition, it poses open-ended questions (e.g., “What concerns you most about today’s world?”) and invites conversations about the exhibition’s themes, offering personalized content (Morena, 2018b). To achieve this in Portuguese, the development team conducted extensive training of Watson with the museum’s data, expanding its vocabulary and preparing it to process open-ended visitor queries. The result is a mediator chatbot that can interpret a variety of questions and respond contextually, connecting visitors with the museum’s ideas in a welcoming tone.

Between 2025 and 2026, for example, the 36th São Paulo Biennial launched IARA (Fundação Bienal de São Paulo, 2025), a virtual guide delivered as a web-based application. As a form of digital mediation, the tool incorporated gamification elements, inviting visitors to recognize artworks through their smartphone cameras, interact with them, and ask questions via a multilingual avatar fluent in Portuguese, English, and Spanish. Presented as an “accessibility experience,” the mediation device was represented by an avatar with dark skin, afro-textured hair, green-framed glasses, and an institutional jumpsuit, helping visitors appreciate 30 selected works from the international exhibition.

This example illustrates that, in Brazil, AI-mediated cultural implementations predominantly rely on screen-based, language-centered systems. While such systems expand access to information and enable personalized content delivery, they offer limited engagement with the spatial and social dimensions that characterize in-person museum experiences. As a result, they tend to rely on interaction models derived from human–computer interaction rather than fully exploring the potential of human–robot interaction in situated cultural environments (Lombard et al., 2024). This limitation becomes particularly salient when museums are understood not merely as exhibition spaces, but as social and affective environments.

Drawing on the Media Are Social Actors (MASA) paradigm—an extension and refinement of the Computers Are Social Actors (CASA) framework proposed by Nass, Steuer, and Tauber (1994); Lombard and Xu (2021) further develop this discussion by arguing that experiences of social presence depend not only on linguistic exchange, but on the extent to which a system embodies characteristics associated with embodied social interaction, such as voice, movement, responsiveness, and physical co-location. From this perspective, technologies that rely predominantly on textual or screen-mediated interactions may have intrinsic limitations in fostering a sustained sense of “being with” another, directly affecting engagement, attention, and meaning-making in mediated encounters. Despite the growing adoption of conversational AI in museums, there remains a lack of empirical studies examining how embodied robotic mediators affect cultural mediation in comparison with chatbots or screen-based systems. In the Brazilian context of museums and cultural institutions, this perspective highlights a significant gap regarding how embodied systems—capable of articulating voice, gesture, spatial orientation, movement, physical presence, and real-time social interaction—may transform the very nature of cultural mediation.

In this context, the present paper, as a situated case study on cultural and educational mediation, examines the development of a proposal that integrates practices from animatronics, creative robotics, and Artificial Intelligence. It offers an opportunity to explore the materialities, practices, and imaginaries involved in the creation of “Zequinha,” a robot conceived as a hybrid device at the intersection of machine, Artificial Intelligence, and puppetry-based artistic practice, and how embodiment can affect the role of AI in cultural institutions. Moreover, this article evaluates “Zequinha” against the six grand challenges to Human-Centered Artificial Intelligence (HCAI) proposed by Garibay et al. (2022): human wellbeing, human-AI interaction, privacy, responsible design of AI, governance and independent oversight, and design and evaluation framework. By situating this investigation within the sociotechnical realities of the Global South, this study aims to highlight how localized, resource-aware, and culturally situated approaches to embodied AI may open alternative pathways for cultural mediation in Brazilian museums and cultural institutions. In doing so, the study seeks ways to investigate how corporeality, physical presence, and multimodal interaction influence cultural mediation practices in museums.

## 2 Materials and methods

The methodology of this study is based on an in-depth case study of the development of the Zequinha robot, positioning it as an experimental field for investigating the incorporation of Artificial Intelligence (AI) into creative processes and cultural mediation. To structure this investigation, a dual framework was adopted: the research-creation approach (Chapman, 2012) as a theoretical–methodological lens, and an iterative, human-centered technical design process as a value-oriented engineering practice.

As an overarching framework, research-creation challenges traditional modes of academic knowledge production by integrating artistic and technological creation as a central pathway for generating knowledge (Chapman, 2012). In this project, the “creation” consists of the development of Zequinha itself—an ongoing act of “directed exploration through creative processes that include experimentation, but also analysis, critique, and deep engagement with theory” (ibid., p. 19). The case study examines how the adoption and adaptation (“tropicalization”) of AI technologies can reconfigure cultural mediation, producing interactive and immersive forms of public engagement.

This objective, however, unfolds into a complex practical challenge: materializing a charismatic and responsive robotic “character” requires the coordinated integration of multiple AI subsystems (language, speech, vision, and movement)—a need that defines this concrete methodological process. Zequinha’s modular architecture—organized as a sequential pipeline of multimodal capture, transcription, intent analysis, language inference, speech synthesis, and movement generation—offered dozens of interchangeable options for each module (hardware, software, and service providers).

Accordingly, the development methodology evolved into an iterative cycle of experimentation and systematic evaluation, guided by three core principles: (1) prioritizing the creation of user comfort, likability, and charisma; (2) seeking a practical balance between performance (e.g., latency and naturalness), cost, and feasibility; and (3) aligning the system with Human-Centered Artificial Intelligence (HCAI) principles, such as local autonomy, privacy, and curatorial control.

## 3 Results and discussion

Through this case study of Zequinha’s development, it was investigated how the adoption of local technologies—such as an SLM like Gemma 3, voice synthesis systems, computer vision, and body movement systems trained for the project—can reconfigure cultural mediation and generate new forms of public engagement that are both interactive and immersive. In contrast to cloud-based LLMs, the local approach affords autonomy, curatorial control, and offline operation, making it a more sustainable solution for cultural institutions.

The development of this project has been shaped by advances in technology and the emergence of artificial intelligence as an accessible tool, as we observe it today. Simultaneously, robots such as Ameca (Engineered Arts), Atlas (Boston Dynamics), ASIMO (Honda), and Unitree H1 (Unitree Robotics) have progressed, driven by well-structured organizations, large teams, and substantial corporate funding—a context distinct from that found in the Global South. References such as the artistic and museological trajectory of Engineered Arts, the robustness of Boston Dynamics’ systems, the longevity of Honda’s research, and Unitree’s cost-accessibility strategy not only serve as inspiration but also help contextualize challenges and reaffirm possible pathways. Accordingly, this work reinforces the idea that it is possible to make an original contribution through proprietary solutions and to demonstrate scientific and technological capability beyond traditional centers, thereby advancing the state of the art.

**FIGURE 1:**
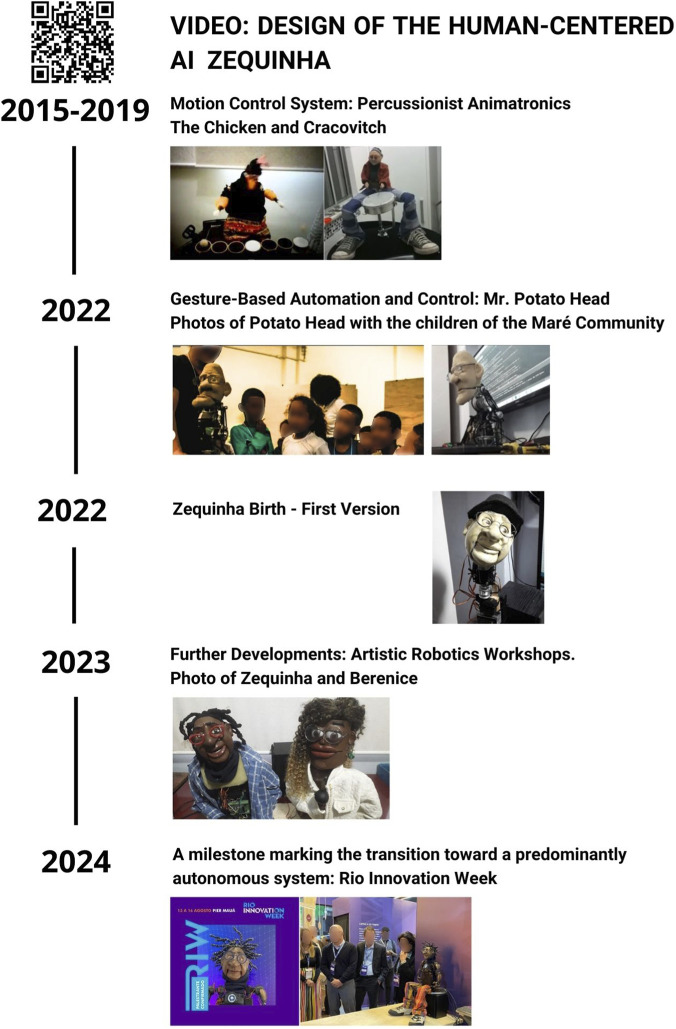
Timeline of the design process of the HCAI robot Zequinha.

Moreover, the project’s trajectory, originating from an inventor with a background in puppetry, reveals a dynamic process of assemblage. The technical architecture that integrates multiple AI modules to create a “character” with customizable behaviors (e.g., friendly, cynical) and the capacity to engage with the public in complex ways. Saulo Dansa, the creator of Zequinha, has a long-standing relationship with puppetry and a deep passion for inventing and integrating technologies. He is the co-founder of Z(i) Arte Tecnologia, an organization dedicated to developing innovative solutions for cultural spaces and science outreach projects, while also conducting art workshops, Artificial Intelligence (AI), and robotics in underserved communities and for deaf individuals. The “birth” of Zequinha occurred within the context of one such workshop, held in two communities in Rio de Janeiro—specifically, the favelas of Morro do Macaco and Morro São João. However, Zequinha was created based on the knowledge and technologies developed through Dansa’s previous projects. Zequinha’s development encompasses five key phases (Figure 1): 1. Animatronic foundations (2015); 2. Transition towards interactivity through more advanced and interactive control systems (2022); 3. Integration of prior learnings and the “birth of Zequinha” (2022); 4. Community cocreation, collaborative evolution, and initial interactions with AI during robotics workshops (2023); 5. The pivotal transition to a predominantly autonomous system employing multimodal AI, triggered by participation in public events (2024).

Zequinha’s foundational animatronics are rooted in the pioneering projects “The Chicken” and “Crakovitch.” These percussionist animatronics established the basis for motion control. Designed as percussion players, they were programmed in the REAPER Digital Audio Workstation (DAW), using the MIDI (Musical Instrument Digital Interface) motion protocol to synchronize movements with the audio track. Motion signals were distributed across separate tracks from the main audio, enabling intuitive and visual synchronization. As a hardware interface, a custom electronic board decoded the MIDI signals and transmitted commands to a microcontroller, which in turn activated the servomotors.

In 2022, the “Potato Head” project marked a transition toward more complex, interactive control systems. This phase introduced stepper motors alongside servomotors. The primary innovation was the development of a gesture-control system using a glove equipped with a gyroscope, accelerometer, and potentiometer. Communication between the glove and the puppet’s microcontroller was wireless, utilizing ESPNOW, a low-latency protocol proprietary to ESP32 microcontrollers.

Zequinha was born at the end of 2022 as a platform designed to integrate the knowledge acquired from the previous projects “The Chicken,” “Crakovitch,” and “Potato Head.” In its initial phase, Zequinha operated without AI, with movements synchronized to musical tracks (such as those of Adoniran Barbosa) through MIDI-based “motion scores.” For fabrication, the head was sculpted manually in clay, followed by silicone molding and casting in polyester resin to produce the final piece. All movable parts were crafted and adjusted by hand. At this stage, an evolution in audio control was also implemented, wherein control signals were mixed directly into the audio track. A C++ programmed microcontroller detected these embedded signals to generate the corresponding movements.

In 2023, a new phase of collaborative evolution and initial AI integration began. Artistic robotics workshops held in the communities of Morro do Macaco and Morro São João facilitated collaboration with children, enabling Zequinha to develop new skills. Control mechanisms were refined with an enhanced glove/joystick, and AI technologies for voice generation and LLM interaction were introduced. In these workshops, the robots Zequinha and Berenice evolved to reflect features suggested by the children, such as dark skin tones, dreadlock-style hair, and glasses.

In 2024, Zequinha reached a pivotal stage of transitioning into a predominantly autonomous system, marked by its invitation from Sesc RJ to participate in Rio Innovation Week (RIW), held from August 13 to 16, 2024. For this event, careful consideration was given to designing the interactive experience for the public, along with the corresponding configurations of the software architecture, control mechanisms, AI integration, and advanced conversational pipelines. Zequinha was programmed to interact with the public daily from 1:00 p.m. to 3:00 p.m. throughout the event. The software architecture used a combination of Python and C++. Moreover, the control involved 12 servomotors operating autonomously under AI guidance. The AI architecture is based on Google Cloud Speech-to-Text (STT) and Text-to-Speech (TTS) for speech-to-text transcription and text-to-speech synthesis, respectively. Moreover, it employed the OpenAI API LLM (ChatGPT) for response generation, TensorFlow and OpenCV for computer vision (including facial recognition and eye tracking), and the Librosa library for lip-sync via analysis of audio waveform amplitudes. The advanced conversational pipeline implemented a complex interaction flow, encompassing user intent detection, question-to-intent mapping, querying local (JSON) or online data sources, conversational history analysis for contextual awareness, and formulation of the final, contextually relevant response.

Zequinha has evolved from a MIDI-programmed animatronic into an autonomous entity that integrates multiple on-device AI systems for real-time interaction, including computer vision, Natural Language Processing (NLP), voice synthesis with lip-sync capabilities, and Spontaneous Movement Generation through a Transformer-based Trained Model (Table 1).

**TABLE 1:** The phases of Zequinha development.

Phase	Year	Movement control	Interaction	Hardware	Software/AI	Conceptual highlights
The chicken and crakovitch	2015 to 2019	MIDI-to-motion mapping via REAPER	Manually choreographed movements, beat by beat, with soundtrack	Handmade built with silicone and polyester resin. PC, custom board, microcontroller (PIC16F84A- microchip technology Inc), mini servo motors	REAPER (DAW), C++	Visual synchronization of movement and sound
Potato head	2022	IMU-based gesture control and audio-level activated robotic mouth controller	Sensorized glove (gesture) and the user’s real-time voice	Foam head, metal structure, and step motors. PC, custom board, drivers for stepmotors, 4 stepmotors (NEMA), glove with gyroscope and accelerometer (MPU-6050), and potentiometer	C++, ESP-NOW protocol (ESP32)	First interactive body interface
Zequinha: first version	Late 2022	Audio command encoding in stereo (audio steganography robotic control)	Choreographed using high-frequency signals embedded in stereo audio	Handmade resin head, PC, ESP32 microcontroller (PIC16F877(A) - microchip technology Inc), 12 servo motors, PWM module PCA9685	C++ (audio signal extraction and motor control)	Manual design and choreographies with music
Zequinha: robotics workshops	2023	Joystick data Input interface + audio feature-based robotic lip-sync	Participatory and collaborative through user audio capture	Joystick-type controller, PC, customized aesthetics by students, and a microphone	Integration with AI-generated voice, LLM, API usage, C++, and python	Cocreation with children, afro-brazilian aesthetic
Zequinha – autonomous (RIW)	2024	Robotic motion control via primitive library + audio feature-based robotic lip-sync	Autonomous conversation with AI and spontaneous gestural movement (still early phase)	PC, microphone, cameras, ESP32, 12 servos, pwm module PCA9685	Python, C++, OpenAI GPT, Google STT, TensorFlow, OpenCV, Librosa, and a diverse ecosystem of libraries	Multimodal pipeline with AI and computer vision

**FIGURE 2:**
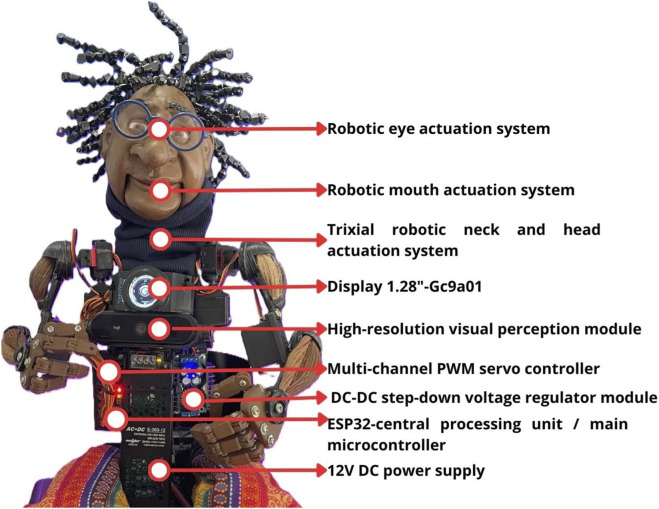
System technical illustration (current version).

With the transition to local AI processing, implemented in 2025, the project’s focus shifted to developing a robust, independent product with an optimized design. The AI models and processes applied in the Zequinha project are summarized in Table 2, and Figure 2 presents the technical illustration of the current version.

**TABLE 2:** Summary of AI models and processes applied in the Zequinha project.

Category	Component/Tool	Description
Embedded AI models	Language processing (LLM)	Gemma 3 (under evaluation) for dialogue generation, context, and reasoning
	Voice synthesis (TTS)	VITS-based model, trained for Zequinha’s custom voice
	Lip sync	VITS component that generates mouth movement parameters from audio
	Speech recognition (STT)	Audio-to-text transcription model
	Spontaneous movement generation	Transformer-based model for contextual body animations
	Facial detection	A model that locates human faces in the camera feed
	Facial recognition (identity)	A model that generates embeddings to identify recurring users
	Facial landmark detection	A model that maps eyes and mouth for tracking
	Facial attribute estimation	A model that estimates age and gender, adapting Zequinha’s performance
	Intention detection	An NLP model that classifies the user’s speech intent
AI engineering	Model optimization (TensorRT)	A model compilation and quantization for optimized use on the Jetson
	Prompt engineering	Creation and management of prompts that shape the personality and responses of the LLM
Supporting AIs	Code assistants	Gemini 2.5 pro, Grok, Deepseek
	Media generation (design)	ChatGPT (DALL-E) for 2D images, Rodin for 3D models

The primary objective centers on embedded processing—executing the entire AI ecosystem, including all inference and control processes, locally, thus eliminating reliance on external APIs and internet connectivity. The local AI models include custom-trained architectures for voice synthesis (based on VITS), lip-sync synchronization, and spontaneous movement generation (using a Transformer-based approach).

For the development of the visual concept, the process incorporated generative image artificial intelligence (DALL-E/ChatGPT), 2D-to-3D conversion via AI (Rodin), refinement using CAD software (ZBrush, Rhinoceros), and final 3D printing in PLA. Thus, AI tools are used in three areas: generating visual concepts and producing technical parts (3D printing); assisting with script generation and architectural discussion; and the tools themselves, installed and trained on Zequinha. For coordination, the AI systems within Zequinha operate in parallel through multithreading. For instance, Zequinha maintains continuous visual tracking even while the microphone is active and data is being transmitted, keeping the “vision” system persistently operational.

This cycle materialized through the active experimentation with multiple configurations, ranging from embedded and edge hardware (Jetson Orin NX – NVIDIA) to distributed cloud infrastructure (Google Cloud). For each configuration, performance metrics—particularly the partial and total latency of the interaction cycle—were rigorously collected through timestamps inserted into Python scripts, enabling direct comparison between, for example, local language models (Gemma 3 4B) and cloud-based APIs (GPT-4o), as well as between local voice synthesis (customized VITS) and service-based solutions (Google TTS). Technical decisions were therefore grounded in empirical evidence regarding their impact on user experience.

In parallel, an ongoing character-centered design process permeated all layers of development. This included manual sculpting of the facial structure in clay, selecting and training a gentle, natural voice model (VITS), defining behavioral traits through prompt engineering, and structuring a “biography” and memory sets in JSON to support identity and contextual grounding. At each software layer, different model architectures and optimization levels were evaluated, consistently seeking the practical threshold at which efficiency would not compromise the quality and naturalness of interaction.

### 3.1 Zequinha’s evaluation under the light of the six HCAI grand challenges

The “Zequinha” Animatronic Robot Project aligns closely with the principles of Human-Centered Artificial Intelligence (HCAI), a framework for developing ethical, equitable, and human-enhancing technologies. Zequinha’s design and development embody several of the six grand HCAI challenges: human wellbeing, responsible design, privacy, design and evaluation framework, governance, and human-AI interaction (Table 3).

**TABLE 3:** The six major challenges of HCAI, considering implemented design decisions and current technical limitations.

HCAI challenge	Design decision	Technical evidence	Main limitation
Human wellbeing	Charismatic and adaptive character design; cocreation with children	Customized voice model (VITS); behavioral modulation; public interaction in events and workshops	Absence of systematic user perception studies
Responsible design	Preference for locally deployed SLM and controlled prompt architecture	Local execution (Gemma 3); structured memory (JSON); personality modulation	Lack of formal bias auditing and explainability tools
Privacy	Embedded processing and offline operation	On-device inference (Jetson); local data storage	Data governance policies are not yet formally consolidated
Design and evaluation framework	Iterative development process (TRL 5–6) and migration to ROS 2	Modular multimodal pipeline; latency measurements; multithreaded architecture	Absence of controlled experimental benchmarking
Governance	Local autonomy strategy and restriction in high-risk domains	Non-deployment in healthcare; institutional control of the AI stack	No formal external ethical oversight
Human–AI interaction	Embodied multimodal architecture	Synchronized lip-sync; eye tracking; parallel vision–speech execution	Lack of quantitative comparison with the chatbot baseline

Central to the project is its emphasis on enhancing human wellbeing. Zequinha was designed to improve visitor experiences in museums and cultural spaces, prioritizing interactive engagement and cultural education at its core. Its development involved collaborative processes, such as artistic robotics workshops with children, during which design elements—like dark skin, dreadlock-style hair, and glasses—were incorporated based on participants’ suggestions. This participatory approach not only promotes inclusivity but also reflects a sensitivity to community-specific cultural representations. Furthermore, Zequinha can estimate the age and gender of its interlocutors and dynamically adjust its behavior, attire, and speech style to suit interactions with different demographics, such as young children or older adults. This adaptive functionality aims to enhance user comfort and minimize frustration, positioning Zequinha not just as an interactive device but as a tool intended to be useful, safe, reliable, and charismatic, with potential applications in the healthcare and public service sectors. Moreover, Zequinha is also used in robotics workshops with deaf people and poor Brazilian communities, fostering participants’ autonomy, robotics competence, and motivation in alignment with the Self-Determination Theory (Ryan and Deci, 2017).

In addressing the challenge of responsible design, Zequinha’s architecture prioritizes transparency and intentionality in its interactions. By relying on locally deployed AI models, such as the SLM Gemma 3, rather than cloud-based LLMs, the project ensures tighter control over content generation, thereby reducing the risk of disseminating inaccurate or misleading information. This choice aligns with curatorial standards that require accurate information and consistent messaging with the institution’s mission. The robot’s behavior can be finely controlled through prompt engineering, allowing designers to modulate its personality along axes such as politeness, cynicism, or warmth, reflecting deliberate character design choices. While broader discussions around bias and accountability in AI are not yet deeply embedded in Zequinha’s current implementation, its use of locally controlled models and datasets represents a foundational step toward addressing such issues.

Privacy is another core consideration in Zequinha’s design. A key project goal has been to localize all AI processing, enabling full functionality without reliance on internet connectivity or external APIs. This approach minimizes the risk of sensitive data being transmitted to third-party servers, safeguarding user privacy. Zequinha’s capacity to store user embeddings locally, alongside conversational data in JSON files, reflects a commitment to institutional data sovereignty, enabling operating organizations to retain control over the information they collect.

Within the design and evaluation framework, Zequinha integrates artistic practice into technological development to support flexible, iterative design processes. Zequinha’s technical evolution—from MIDI-programmed animatronic systems to a multimodal AI-driven platform—illustrates this phased and responsive approach. To manage increasing system complexity, the project has adopted the Robot Operating System 2 (ROS 2), which facilitates code maintenance, scalability, and rapid deployment of new functionality, ensuring the platform’s long-term viability. It is interesting to contrast Zequinha’s development methodology with the HCI Double Diamond process presented by Ozmen Garibay et al. (2023). Zequinha’s design process was not linear but a continuous, fluid, iterative improvement process, based on trial-and-error and users’ experience. Furthermore, human and societal wellbeing are at the center of the design process, as described previously, in which human participants were involved in co-creation.

In terms of governance and independent oversight, Zequinha’s development embodies principles of local autonomy and adaptive innovation, reflecting the project team’s strategy of pursuing offline operability and resource-efficient solutions in response to chronic underfunding in Brazil’s innovation landscape. Strategic decisions—such as refraining from deploying Zequinha in healthcare contexts until the surrounding ethical and community discussions mature—underscore a deliberate approach to responsible AI deployment that prioritizes social readiness over mere technical capability. Moreover, independent oversight receives special attention in cases where AI has broad use (Ozmen Garibay et al., 2023), which is not the case with Zequinha.

Finally, Zequinha has been designed with respect for human cognitive processes. Its architecture employs multithreading to enable the parallel operation of subsystems, such as computer vision and audio processing, allowing the robot to maintain eye contact while simultaneously listening and preparing responses, thereby promoting fluid, human-like interactions. The integration of VITS-based speech synthesis with precise lip synchronization and spontaneous body movements generated through transformer models further enhances the naturalness and comprehensibility of the interaction. Training these physical movements concerning conversational context exemplifies the project’s commitment to designing AI systems that complement, rather than disrupt, human communication. Zequinha’s contextual awareness—its ability to analyze conversation history, access local databases, and retrieve up-to-date information from the web—further enables meaningful, low-friction interactions that respect users’ cognitive load and facilitate efficient communication. Considering the factors highlighted by Mitchell and Jeon (2025) to enhance HRI, Zequinha is a good example of a charismatic design that focuses on identity (the unique characteristics of a robot through naming and personality traits) and effective communication (through speech, gestures, or eye contact) to create connections with users.

In summary, the Zequinha project embodies an effort to integrate artificial intelligence within human-centered frameworks, thereby enhancing human capacities and enriching cultural and educational experiences while upholding autonomy, safety, and adaptability as foundational principles. By contextualizing its technological innovations within the realities of Brazilian socioeconomic conditions, Zequinha exemplifies the potential of localized, ethically-informed AI development that bridges technology, art, and community engagement.

## 4 Limitations and future studies

Given the limitations of this work, future studies could empirically examine participants’ perceptions in the museum in greater detail. For example, surveys could be conducted and triangulated with observation, presence data, and document analysis.

This paper focuses on the notion of the “embodiment” of AI—specifically, within the Brazilian context, the fusion of artificial intelligence with creative robotics as part of a process we describe as the “tropicalization of technologies.” In Brazil, there exists a long-standing tradition of improvised technological practices, known as “gambiarras”, “geringonças”, and “engenhocas” (Paulino and Mafra, 2012). This is a cultural practice of solving problems in alternative ways, cobbling parts together, and offering unusual, improvised solutions. The work of Fred Paulino, Ganso, and Lucas Mafra (Paulino and Mafra, 2008), who introduced and theorized the concept of “gambiology”, offers a relevant cultural and theoretical framework for analyzing the development of the Zequinha robot. As a contemporary reinterpretation of “gambiarra”, gambiology investigates the Brazilian tradition of improvising and creating solutions from available resources, combining art, design, and technology within the realm of digital culture (Boufleur, 2013; Gontijo, 2014; Lopes, 2019).

In this context, concepts such as “gambiarra”, Human-Centered Artificial Intelligence (HCAI), the Global South, and the “tropicalization” of technologies converge, highlighting adaptive creativity not as a circumstantial response but as a foundational methodology for developing innovation under conditions of structural constraint. From this perspective, the Zequinha project can be interpreted as offering a tropicalized, art-driven model of HCAI.

Zequinha’s technical development unfolded incrementally and through hybridization. The robot did not emerge as a complete, predefined system; instead, it evolved in stages, progressively integrating new technologies. The process of tropicalizing technology and responding to scarcity is also key to understanding Zequinha’s development. Discussions about the use of a local SLM rather than cloud-based LLMs, and the acknowledgement of Brazil’s structural lack of suitable funding mechanisms, highlight the broader sociotechnical context. Within this scenario, gambiology emerges as a creative response to resource scarcity, where the pursuit of autonomy and offline operation reflects a deliberate strategy of technological “tropicalization”—adapting innovation to local realities rather than depending on external infrastructures.

In this article, the practices referred to as “gambiarras” are not understood as substitutes for engineering rigor, but rather as research-creation strategies situated at intermediate stages of technological development. The development of Zequinha follows an incremental trajectory typical of robotics projects. Positioned between levels 5 and 6 of the Technology Readiness Level (TRL) scale (Mankins, 2004)—corresponding to validation in a relevant environment and demonstration in an operational context—the project has prioritized validating core interaction capabilities and building a functional multimodal pipeline. In this context, migration to Robot Operating System 2 (ROS 2), a framework directly aligned with scalability objectives, is already underway. Subsequent iterations, targeting higher maturity levels (TRL 7+), focus on product-level attributes such as robust safety protocols, extensive reliability testing, and scalable architectures—objectives that ROS 2 itself was designed to support and which constitute the focus of the current research and development phase.

Several challenges remain in the development of Zequinha, including the training process, the simultaneous execution of multiple AI systems, safety systems, and human–robot interaction, and funding constraints. The first challenge concerns AI implementation, as the training process is complex and requires large datasets and extensive processing time. Another significant challenge lies in enabling the low-latency, simultaneous execution of multiple AI systems to support fluid conversation, which demands substantial computational effort in terms of processing power and architectural design. A third aspect concerns safety systems and human–robot interaction.

Finally, Brazil lacks adequate funding mechanisms for projects of this nature, posing a significant barrier to implementation. The scarcity of financial resources allocated to initiatives of this type highlights a gap in public policies supporting the sector. The Zequinha project is a robust, continuously evolving platform for research and development in human–robot interaction. Its transition to a local AI ecosystem demonstrates its viability as an autonomous product, through which we seek to integrate art and technology. Zequinha exemplifies the flexibility and adaptability of AI technologies, which can be applied across diverse contexts—from museums and cultural institutions to healthcare and industry.

To further advance research and creative development with Zequinha, we are considering a “tech-together” strategy. This adaptable framework may encompass partnerships, consultancy, and infrastructure support (advisory services, processing time, and the use of platforms such as Google Colab), alongside targeted institutional collaborations with cultural organizations or international research centers. Our goal is to identify methodological insights that can be systematically documented and replicated.

## Data Availability

The original contributions presented in the study are included in the article/Supplementary Material, further inquiries can be directed to the corresponding author.
