# Supplementary material for: Bridging art and AI in the global south: the development of the robot Zequinha considering the grand challenges of human-centered artificial intelligence
Source: Front Robot AI. 2026 Feb 25;13:1765950. doi: 10.3389/frobt.2026.1765950 (PMC12975421; doi:10.3389/frobt.2026.1765950)
Supplement: Supplementary file 1 [file Supplementaryfile1.docx]

**HARDWARE SPECIFICATIONS AND ADDITIONAL TECHNICAL DETAILS**

▬▬▬▬▬▬▬▬▬▬▬▬▬▬▬▬▬▬▬▬▬▬▬▬▬
**NVIDIA Jetson Orin NX**

System-on-Module (SoM) for edge computing, designed for the local execution of deep learning models and complex computer vision pipelines in robotics and autonomous devices.

CORE SPECIFICATIONS
1. CPU Cores: 8 physical cores based on the Arm Cortex-A78AE architecture, organized as 6 high-performance cores plus a 2-core high-efficiency cluster.
2. GPU CUDA Cores: 1024 cores based on the NVIDIA Ampere architecture (GPU), enabling parallel general-purpose computation (CUDA, OpenCL).
3. Tensor Cores: 32 third-generation (Ampere) cores specialized for matrix operations (AI/Deep Learning – FP16, INT8, etc.).
4. RT Cores: 16 second-generation (Ampere) cores dedicated to hardware-accelerated ray tracing.

TOTAL CORE COUNT: 1080 cores (8 CPU + 1024 CUDA + 32 Tensor + 16 RT).
Power Consumption: 10-25W.
▬▬▬▬▬▬▬▬▬▬▬▬▬▬▬▬▬▬▬▬▬▬▬▬▬
**RASPBERRY PI 5**

General-purpose Single-Board Computer (SBC).
Based on the Broadcom BCM2712 SoC, featuring a quad-core Arm Cortex-A76 CPU, VideoCore VII GPU, and 8 GB of LPDDR4X memory. It provides robust peripheral connectivity, including PCIe 2.0 and USB 3.0 interfaces. The platform offers a cost-effective solution for automating tasks and executing software applications and lightweight AI models.
Power Consumption: 5-10W.
▬▬▬▬▬▬▬▬▬▬▬▬▬▬▬▬▬▬▬▬▬▬▬▬▬
**ESP32**Microcontroller designed for low-power applications.

Core Architecture: System-on-Chip (SoC) featuring a dual-core Xtensa LX6 processor operating at up to 240 MHz.
Native Wireless Connectivity: Integrates on-chip Wi-Fi 802.11 b/g/n (2.4 GHz) and Bluetooth/Bluetooth Low Energy (BLE) 4.2 transceivers.
ESP-NOW Protocol: Proprietary communication protocol enabling direct transmission of small data packets (up to 250 bytes) between ESP32 devices without requiring Wi-Fi infrastructure. It provides low-latency communication, supports peer-to-peer and one-to-many topologies, and offers optional encryption.
Peripherals and I/O: Includes versatile GPIOs and multiple communication interfaces, including SPI, I2C, I2S, UART, and ADC. Additionally, it features an Ultra-Low-Power (ULP) coprocessor for sensor monitoring during deep sleep operation.

Power Consumption: Supports low-power operating modes (e.g., deep sleep) with current consumption in the microampere (µA) range.
▬▬▬▬▬▬▬▬▬▬▬▬▬▬▬▬▬▬▬▬▬▬▬▬▬

**GEMMA 3**gemma-3-4b-it-Q4_K_M.gguf

AI model optimized for efficiency.
Base Model: Gemma 3 with 4 billion parameters, instruction-tuned (-it variant) to enhance performance in instruction-following tasks.
Quantization: Compressed to 4-bit precision (Q4) using the K-quants (K_M) method, providing an effective trade-off between model quality and memory footprint.
Purpose: Well-suited for deployment in embedded systems, such as robotic platforms operating on CPUs or GPUs with limited RAM, enabling offline natural language reasoning with low computational overhead.

Memory Footprint: Approximately 2.5–3.5 GB.
▬▬▬▬▬▬▬▬▬▬▬▬▬▬▬▬▬▬▬▬▬▬▬▬▬
**ARCHITECTURE**The system architecture is organized as a modular, sequential pipeline distributed between the client (the physical agent Zequinha) and one or more processing servers. The complete interaction workflow comprises the following stages:

**Phase 1 – Client (Acquisition and Actuation):**Simultaneous acquisition of audio (via microphone) and video (via camera).
Local visual processing for facial tracking, extraction of facial coordinates, and eye-movement control.
Packaging and transmission of audio streams and video frames to the server over the network.

**Phase 2 – Server (Cognitive Processing and Generation):**Transcription and Visual Analysis: Automatic Speech Recognition (ASR) and extraction of user attributes (e.g., approximate age).
Intent Detection: Inference using a Small Language Model (SLM) to classify communicative intent..
Dynamic Contextualization: Automated prompt construction integrating conversation history, user profile, the agent’s persistent memory, and the detected intent.
Response Generation: Inference using a language model (SLM/LLM) to generate a contextually grounded textual response.

Multimodal Response Synthesis:
Speech Synthesis (TTS): Conversion of the generated text into natural speech.
Lip-Sync Data Generation: Production of lip-synchronization parameters to ensure alignment between speech and facial articulation.
Body Motion Generation: Generation of body movement sequences using a Transformer-based model.

**Phase 3 – Return and Execution:**Transmission of the synthesized data (audio, lip-sync parameters, and motion sequences) back to the client.
Reception and execution by the physical agent: audio playback, actuation of servomotors, and synchronization of movements with speech.

**Architectural Characteristics:**Modularity: Each stage of the pipeline is interchangeable, enabling experimentation with different hardware platforms, models, and service providers.
Flexible Deployment: Processing can be performed locally (on the client or on a nearby server) or distributed in the cloud, with direct implications for system latency.
Asynchronous Communication: The system accommodates variable network latencies, ranging from local scenarios (< 5 ms) to geographically distributed configurations (> 200 ms).
This architecture underpins the research-creation methodology, as the multiplicity of options within each module creates a broad combinatorial design space that requires systematic experimentation to optimize performance, cost, and interaction quality.

▬▬▬▬▬▬▬▬▬▬▬▬▬▬▬▬▬▬▬▬▬▬▬▬▬

**LATENCIES:**

Data acquisition for comparative latency and performance studies was conducted by inserting timestamps at multiple stages of the Python scripts, enabling the measurement of actual processing times and end-to-end latencies for each pipeline component. Performance reports and analytical assessments were generated using averaged empirical values, accounting for real-world variability.
Based on the quantification of these latencies, new strategies and alternative architectural configurations are iteratively implemented to achieve more fluid and engaging communication between the robot (Zequinha) and the user. This remains an ongoing process of research and experimentation.

Table 1: Technical Parameters of Network Latency

Comparative Latency Metrics in the Zequinha Pipeline

SAMPLING

==========================================================
Categories Configuration Model/Provider Latency(ms)
==========================================================

Transcription (STT) / Speech Recognition
—-------------------------------------------------------------------------------------------------
 Local Whisper-tiny 1,487.68
 Local Whisper-base 829.27
 API (cloud) Whisper API 1,331.00
 API (cloud) Google Speech 1,672.84
—-------------------------------------------------------------------------------------------------
Inference (LLM) / Response Generation —-------------------------------------------------------------------------------------------------
 Local Gemma-3-4B 3,265.42
 API (cloud) GPT-4o-mini 1,775.70
 API (cloud) GPT-4o 1,268.03
 API (cloud) GPT-3.5-turbo 789.47
—-------------------------------------------------------------------------------------------------
Speech Synthesis (TTS) / Audio Generation —-------------------------------------------------------------------------------------------------
 Local VITS-custom 147.45
 API (cloud) Google TTS 2,058.22
—-------------------------------------------------------------------------------------------------

Table 2: Technical Parameters of Network Latency Across the Tested Topologies

SAMPLING

========================================================
INTRA-HOST COMMUNICATION (SAME MACHINE)
-------------------------------------------------------------------------------------------------
Processes on the Same PC 0.1 ms
-------------------------------------------------------------------------------------------------
WIRED LOCAL AREA NETWORK (LAN) COMMUNICATION
-------------------------------------------------------------------------------------------------
PC <-> ESP32 (via Ethernet) 0.1- 3 ms
PC <-> Jetson Orin NX 0.1 - 5 ms
PC <-> Raspberry Pi 5 0.1 - 5 ms
-------------------------------------------------------------------------------------------------
COMMUNICATION VIA CLOUD INFRASTRUCTURE (WAN)
-------------------------------------------------------------------------------------------------
Host Local (PC/RPi5/Jetson) <-> VM Regional (São Paulo) 6 - 35 ms
Host Local (PC/RPi5/Jetson) <-> VM USA (Iowa) 110 - 200 ms
Host Local (PC/RPi5/Jetson) <-> VM Europe (Frankfurt) 180 - 290 ms
========================================================

Note: Constructed using identical parameters across all stages of the pipeline.

Parameters:
Prompt used for transcription: “Olá, tudo bem, como vai você?”
*Audio duration: 2 s
Number of tokens (input): 10
Language: Portuguese
Max_tokens/response: 100
Temperature = 0.5*Reference response used for TTS sampling: *Olá! Estou bem, obrigado por perguntar. E você, como está?*

Sample Configuration: *Local Hardware*
- CPU: AMD Ryzen 7 5700G (16 cores, 4.6 GHz max)
- GPU: NVIDIA GeForce RTX 3060 (12GB VRAM)
- RAM: 64GB DDR4
- Storage: NVMe SSD 500 GB

Software:
Operating System (OS): Ubuntu 24.04 LTS (Linux kernel 6.14.0-37-generic, optimized for inference).
- Python: 3.13.11
- CUDA: Driver 570.158.01

▬▬▬▬▬▬▬▬▬▬▬▬▬▬▬▬▬▬▬▬▬▬▬▬▬

**LIBRARIES**
A wide range of libraries have been evaluated throughout iterative performance optimization efforts, including:
PyTorch com suporte CUDA, Whisper, llama.cpp, CV2, pydub, pyautogui, time, threading, mediapipe, audioop, multiprocessing, numpy, openai, os, pygame, librosa, speech_recognition, service_account, re, soundfile, queue, random, gender_guesser.detector, winsound, datetime, glob, json, tkinter, atexit, subprocess, transformers, torch, tensorflow, dlib, sounddevice, pyaudio, whisper, TTS, gTTS, gpiozero, RPi.GPIO, pyserial, asyncio, requests, logging, pickle, psutil, matplotlib,torch, tensorflow-gpu, jax[cuda], cupy, numba, pycuda, pytorch3d, transformers[torch]
▬▬▬▬▬▬▬▬▬▬▬▬▬▬▬▬▬▬▬▬▬▬▬▬▬
**SERVO MOTORS: MG996R**Signal Frequency: 50 Hz, Angular Range: 270°, Torque: 9.4 kgf·cm at 4.8V; 11 kgf·cm at 6V.
Speed: 0.17 s/60° at 4.8V; 0.14 s/60° at 6V.
Control Signal: PWM - Gear Type: Metal gears - Connector: Standard JR-type.
Operating Voltage: 6V.
Idle Current: ~10 mA. Operating Current (under load): 500 mA to 1.5A.
